# A comparison of five methods for selecting tagging single-nucleotide polymorphisms

**DOI:** 10.1186/1471-2156-6-S1-S71

**Published:** 2005-12-30

**Authors:** Kelly M Burkett, Mercedeh Ghadessi, Brad McNeney, Jinko Graham, Denise Daley

**Affiliations:** 1 The James Hogg-iCAPTURE Centre for Cardiovascular and Pulmonary Research, University of British Columbia, St. Paul's Hospital, Vancouver, BC V6Z 146, Canada; 2 Department of Statistics and Actuarial Science, Simon Fraser University, Burnaby, BC 15A 156, Canada; 3 Department of Epidemiology and Biostatistics, Case Western Reserve University, 44106, Cleveland, OH, USA

## Abstract

Our goal was to compare methods for tagging single-nucleotide polymorphisms (tagSNPs) with respect to the power to detect disease association under differing haplotype-disease association models. We were also interested in the effect that SNP selection samples, consisting of either cases, controls, or a mixture, would have on power. We investigated five previously described algorithms for choosing tagSNPS: two that picked SNPs based on haplotype structure (Chapman-haplotypic and Stram), two that picked SNPs based on pair-wise allelic association (Chapman-allelic and Cousin), and one control method that chose equally spaced SNPs (Zhai). In two disease-associated regions from the Genetic Analysis Workshop 14 simulated data, we tested the association between tagSNP genotype and disease over the tagSNP sets chosen by each method for each sampling scheme. This was repeated for 100 replicates to estimate power. The two allelic methods chose essentially all SNPs in the region and had nearly optimal power. The two haplotypic methods chose about half as many SNPs. The haplotypic methods had poor performance compared to the allelic methods in both regions. We expected an improvement in power when the selection sample contained cases; however, there was only moderate variation in power between the sampling approaches for each method. Finally, when compared to the haplotypic methods, the reference method performed as well or worse in the region with ancestral disease haplotype structure.

## Background

Case-control designs are increasingly used in candidate gene association studies to detect common disease alleles. Traditionally, this design requires an *a priori *hypothesis of the genes to be tested for association. A key concept underlying the design of any disease-marker association study is linkage disequlibrium (LD), or the nonrandom assortment of alleles. LD can be used to identify single-nucleotide polymorphisms (SNPs) that efficiently represent other SNPs in a given region; these SNPs have been called tagging SNPs (tagSNPs). The goal is to select tagSNPs in order to reduce genotyping costs without losing the ability to detect disease associations. Many methods have been developed for selecting tagSNPs, using criteria such as haplotype diversity and pairwise LD. Obtaining samples from sources such as the HapMap  for SNP discovery and LD or haplotype characterization can save both time and genotyping costs, but may compromise power. If a disease allele is rare it may be optimal to sample a population of cases to select tagSNPs, rather than a sample consisting only of healthy individuals.

We assessed the performance of five methods: Stram et al.(implemented in TAGSNPS), Chapman et al. (haplotypic and allelic, implemented in HTSNP2), Cousin et al. [1-3], and the recently proposed approach of Zhai et al. [4] as a control method. We will simply refer to these as the Stram, Chapman-haplotypic, Chapman-allelic, Cousin, or Zhai methods, respectively. TagSNPs were chosen from an initial sample of cases-only, controls-only, and a combined case/control sample in two regions with known disease association. We estimated the power of the tagSNP sets to detect association over 100 simulated case-control studies and compared the number of tagSNPs selected. Although tagSNP methods have been assessed and compared, little information is available on how well the methods compare under different haplotype-disease association models, and the effect that sampling population has on tagSNP selection.

## Methods

Performance of the tagSNP selection methods was determined by comparing the results of case-control association studies. Using the Genetic Analysis Workshop 14 simulated dataset and answers, we selected 2 candidate regions for analysis: D2 and D4. We chose these regions because they were known to contain a disease locus and were simulated to have differing haplotype-disease association structure. Region D2 was simulated with the disease allele inserted into structurally similar haplotypes, mimicking the case of a mutation arising on an ancestral haplotype. Region D4 was simulated with the disease allele inserted into haplotypes of similar frequency so that the disease mutation was not tied to haplotype structure. In practice one would select SNPs flanking the region of interest, and so we included 5 SNPs on both sides of our regions, except for region D2, which is at the right end of the chromosome. The microsatellite locus D09S0348 in region D4 was removed. We considered 17 SNPs in the D2 region and 22 SNPs in the D4 region.

We assessed the performance of 4 tagSNP methods that we classify as allelic or haplotypic and a fifth method that we use as a control (Zhai). In allelic (Cousin and Chapman-allelic) or single-SNP approaches, a SNP is a tagSNP if it is a good surrogate for other SNPs based on some pair-wise measure such as LD or power to detect an association. In haplotypic approaches (Stram and Chapman-haplotypic), the set of tagSNPs captures the information on the haplotype structure in the region.

Stram's method [1], motivated by the common-disease, common-haplotype hypothesis, seeks to identify tagSNP haplotypes that predict common haplotypes by maximizing the minimum coefficient of determination for common haplotypes, R_h_^2^. The minimum R_h_^2 ^is maximized over all possible tagSNP subsets of a given size. Chapman's implementations (allelic and haplotypic) [2] assume a single causal locus in the region, whose alleles may be predicted by haplotypes of tagSNPs (haplotypic), or tagSNP alleles (allelic). The association between tagSNP alleles or haplotypes and the causal locus is measured through the coefficient of determination, *R*^2^, under the assumption that predicting the true causal locus is no more difficult than predicting any of the SNPs in the region. Cousin's method [3] selects tagSNPs that maximize the power of detecting association with an unobserved disease locus in LD with SNPs in the set. The power of a set is found by averaging over defined disease model penetrances and over each SNP in the candidate region, assuming each such SNP has an equal chance of being the susceptibility locus. Finally, Zhai's method [4] selects *k *tagSNPs as equally spaced throughout the candidate region as possible. This is achieved by selecting tagSNPs that minimize the variance of pair-wise SNP distances, as measured on the linkage map. The description of the method does not include criteria for choosing *k*; therefore, we use it as a control method to verify that the other tagSNP methods actually offer improvements over this more intuitive approach.

For Stram's method, we set the minimum haplotype frequency cut-off to 0.04. Chapman's method was run using a minor allele frequency cut-off of 0. Both Stram and Chapman use an *R*^2 ^parameter that measures the coefficient of determination for the underlying model and in both cases we set this parameter to 0.80. We implemented Cousin's method as described in the paper since no software was available. For these 4 methods, subset size was increased until the corresponding thresholds of R_h_^2^, *R*^2^, and maximal power were attained. We used threshold values given in the original papers. Our implementation of Zhai's method utilized the number of tagSNPs selected by both the Chapman-haplotypic and Stram method as the value of *k*, and selected from all SNPs. The best set of tagSNPs was chosen from among 10^6 ^randomly generated candidate sets.

For tagSNP selection, we randomly selected 24 cases, 24 controls and an equal mixture of 24 cases and controls from the entire population. After tagSNP selection, we performed a case/control association study using 100 cases and 100 controls. Initially, 50 samples were used for tagSNP selection and 500 cases and 500 controls were chosen for the association study. However, we found that the association was too strong to allow meaningful differentiation of the methods, so sample size was lowered. Cases and controls in the association study were randomly selected from the Karangar datasets and included individuals from the tagSNP selection step. Single-locus *p*-values were obtained from chi-square tests of allelic association. The most significant (i.e., minimum) Bonferroni-corrected *p*-value within a candidate region and the number of tagSNPs selected were recorded for each method. We repeated this experiment with 100 random samples and estimated power with the proportion of replicates having the Bonferroni-corrected *p*-value less than 0.05. We determined that differences greater than 10% are greater than simulation error and therefore considered these noteworthy (calculations not shown). Because the allelic test assumes Hardy-Weinberg equilibrium (HWE), we tested for HWE in all SNPs across replicates and found no evidence for deviation at the 5% level after correcting for multiple tests in both regions (results not shown).

## Results and Discussion

Although there was consistency over the 100 replicates in the number of tagSNPs chosen by a given method, there were considerable differences across methods in the number of tagSNPs selected (see Table 1). Cousin and Chapman-allelic select nearly all SNPs in both candidate regions as tagSNPs. Since these methods are dependent on the presence of pair-wise LD, we looked at allelic correlations (*r*^2^) in both regions in our first two replicates and found unexpectedly low levels of pair-wise LD. In contrast, the haplotypic approaches of Stram and Chapman selected half as many tagSNPs as the non-haplotypic approaches in both regions. On average, Stram chose one more SNP than Chapman-haplotypic. In comparing the SNP sets selected by Stram and Chapman-haplotypic, we found that the average proportion of SNPs in common, relative to the number of all SNPs chosen by both methods, was approximately 30% (results not shown). Cousin and Chapman-allelic choose almost all SNPs, and on average 94% of SNPs were shared in common (results not shown).

Estimated power across all methods was higher in the D2 region than in the D4 region, likely reflecting the underlying disease models used in the data simulation. The estimated powers of Cousin and Chapman-allelic were essentially equal in D2 and D4, and were generally higher than those of the haplotypic methods. Since these methods chose nearly all the SNPs in the region, they basically give the underlying power to detect association The haplotypic method of Stram had approximately 10% lower estimated power in the D2 region than the allelic methods. The estimated power of Chapman-haplotypic in the D2 region was consistently lower than that of Stram across tagSNP sample sets, but was within the 10% simulation error range. In D4, Stram had estimated power within 10% of the allelic methods. On the other hand, Chapman-haplotypic had greater than 10% differences in estimated power relative to the allelic methods. However, Chapman-haplotypic was within 10% of Stram, except in the cases sample, where there was a 16% reduction in estimated power relative to Stram. Generally, power was estimated to be higher for the allelic methods than for the haplotypic methods, indicating that even if there is sufficient haplotypic structure to reduce the tagSNP set size, this may result in a loss of power to detect association.

By choosing equidistant SNPs, Zhai's method is similar to the SNP selection approach one might use in practice. Zhai et al. [4] concluded that choosing equally spaced SNPs performed as well as the HapBlock method [5] that selects tagSNPs based on haplotype blocks. For each replicate, we chose the subset sizes for Zhai to match the number of SNPs chosen by both Chapman-haplotypic and Stram. Because Cousin and Chapman-allelic chose almost all SNPs in each region, the comparison to Zhai's method would not be meaningful and would be expected to have the same power. In the D2 region, Zhai had at least 12% less power than Stram for SNP subsets of equal size. However, in the D4 region differences between Zhai and Stram were within 10%. This could suggest that choosing SNPs to tag common haplotypes offers increased power if in the candidate region similar haplotypes carry the disease locus. Alternatively the poor performance of Zhai in D2 may be because the hidden disease locus was located at the very end of the D2 region. Our implementation of Zhai's method cannot select the last SNP in a region, and because we were unable to pad D2 with extra SNPs at the disease-locus end, a potentially important disease-associated SNP could be missed. We re-implemented Zhai to force the inclusion of the last SNP in the region into the tagSNP set, but this did not improve estimated power (results not shown). In contrast, Zhai performed about as well (in D2) or better (in D4) than Chapman-haplotypic.

We had hypothesized power would increase when the tagSNP selection sample contained cases only, because cases would be more likely to carry disease haplotypes. However, the power for the control samples was often greater than or equal to that of the cases. With only moderate variations under 7% in estimated power between the different tagSNP sampling approaches within each method, the variation is within simulation error and we cannot conclude that the initial tagSNP sample altered power.

## Conclusion

Our motivation for this study was to compare different methods and sample populations for tagSNP selection with respect to the power to detect disease association. We found that there were no significant differences in estimated power between the 3 selection samples. However, we do note that in regions of low pair-wise LD, reducing the number of SNPs genotyped appears to reduce the power to detect an association, as seen by the generally poorer performance of the smaller tagSNP sets from the haplotypic approaches. Larger samples would have to be recruited in order to offset this lower power. Although we did not determine which thresholds were optimal, for haplotypic methods the suggested thresholds of 0.8 for *R*^2^-values may yield tagSNP sets underpowered to detect association. Those using these approaches should consider larger *R*^2 ^thresholds. Finally, we did not replicate the findings of Zhai et al. [4] that tagSNP subsets were no better than equally spaced SNP subsets. In the D2 region, we found that the Stram method had better estimated power than the Zhai method.

There are a few points that limit generalization of these results that we did not address because of time and computational limitations. For example, we could have compared power across methods after forcing the methods to select equal numbers of tagSNPs. Without equal numbers of SNPs, it is unclear whether any differences in estimated power are due simply to the size of the tagSNP set rather than the methods examined. However, for Stram in D2 there was a clear improvement over tagSNP sets of the same size with equally spaced SNPs. Hence, in some situations tagSNP methods can capture more information than a reasonable SNP subset size. Additionally, our study used simulated data. While these data were based on real data from chromosome 6, the methods used to simulate the disease alleles may not reflect what actually occurs in nature. The regions we examined contained low levels of pair-wise LD, and in practice one may not actually use a tagSNP selection strategy in such regions because of the potential to miss a true disease locus.

## Abbreviations

HWE: Hardy-Weinberg equilibrium

LD: Linkage disequilibrium

SNP: Single-nucleotide polymorphisms

tagSNPs: Tagging SNP

## Authors' contributions

Design of study and research question: KMB, DD, MG, JG, BM. Implementation of new methods: KMB, JG, BM. Running methods: KMB, DD, MG. Writing manuscript: KMB, DD, MG. Editing and proofreading manuscript: KMB, DD, MG, JG, BM. All authors read and approved the final manuscript.

## Figures and Tables

**Table 1:** Summary of *p*-values, estimated power and size of tagSNP sets over 100 replicates

		D2 Region			D4 Region		
			
Method	Sample	Median *p*-value^a ^[1^st^, 3^rd ^quartile]	Estimated power^b ^(SE)	Mean # tagSNPs (SD)	Median *p*-value^a ^[1^st^, 3^rd ^quartile]	Estimated power^b ^(SE)	Mean # tagSNPs (SD)
Cousin	cases	0.034 [0.0002, 0.0173]	0.88 (0.033)	16.5 (0.6)	0.050 [0.0131, 0.1043]	0.49 (0.051)	20.2 (0.9)
	controls	0.021 [0.0002, 0.0173]	0.88 (0.033)	16.6 (0.5)	0.041 [0.0134, 0.1113]	0.53 (0.050)	20.7 (0.8)
	mixture	0.033 [0.0002, 0.0176]	0.87 (0.034)	16.5 (0.6)	0.040 [0.0125, 0.1124]	0.55 (0.050)	20.7 (0.7)

Chapman-allelic	cases	0.002 [0.0002, 0.0176]	0.88 (0.033)	16.9 (0.3)	0.050 [0.0129, 0.2307]	0.50 (0.050)	20.0 (1.0)
	controls	0.002 [0.0002, 0.0176]	0.88 (0.033)	16.8 (0.4)	0.047 [0.0136, 0.1792]	0.50 (0.050)	20.8 (0.8)
	mixture	0.002 [0.0002, 0.0176]	0.88 (0.033)	16.9 (0.4)	0.041 [0.0135, 0.1261]	0.52 (0.050)	20.5 (0.9)

Stram	cases	0.013 [0.0032, 0.0505]	0.73 (0.044)	7.8 (0.6)	0.042 [0.0101, 0.2754]	0.53 (0.050)	8.1 (1.0)
	controls	0.003 [9 × 10^-5^, 0.0348]	0.76 (0.043)	7.9 (0.7)	0.069 [0.0066, 0.2745]	0.44 (0.050)	8.0 (1.1)
	mixture	0.008 [4 × 10^-4^, 0.0769]	0.70 (0.046)	7.7 (0.6)	0.075 [0.0133, 0.2368]	0.46 (0.050)	7.9 (1.0)

Zhai^c ^(Stram)	cases	0.039 [0.0126, 0.146]	0.59 (0.049)		0.099 [0.0141, 0.2564]	0.44 (0.050)	
	controls	0.039 [0.0088, 0.1642]	0.59 (0.049)		0.080 [0.0132, 0.2562]	0.46 (0.050)	
	mixture	0.037 [0.0112, 0.1538]	0.58 (0.049)		0.059 [0.0124, 0.2565]	0.50 (0.050)	

Chapman-haplotypic	cases	0.022 [0.0052, 0.1553]	0.64 (0.048)	6.9 (0.4)	0.150 [0.0143, 0.5386]	0.37 (0.048)	7.6 (0.6)
	controls	0.011 [0.0006, 0.1126]	0.68 (0.047)	6.8 (0.4)	0.105 [0.0197, 0.3639]	0.37 (0.048)	7.5 (0.6)
	mixture	0.016 [0.0014, 0.0970]	0.66 (0.047)	6.9 (0.5)	0.115 [0.0152, 0.4258]	0.41 (0.049)	7.5 (0.6)

Zhai^d ^(Chapman haplotypic)	cases	0.041 [0.0116, 0.1805]	0.61 (0.049)		0.048 [0.0103, 0.2322]	0.50 (0.050)	
	controls	0.022 [0.0060, 0.1126]	0.67 (0.047)		0.055 [0.0108, 0.2196]	0.49 (0.050)	
	mixture	0.037 [0.0086, 0.1620]	0.62 (0.049)		0.059 [0.0107, 0.2192]	0.48 (0.050)	

^a^The median of the Bonferroni-corrected *p*-values from 100 replicates.

^b^The proportion of replicates with Bonferroni-corrected *p*-value < 0.05.

^c^The number of tagSNPs chosen for Zhai is set to be the same as Stram for each sample within replicate.

^d^The number of tagSNPs chosen for Zhai is set to be the same as Chapman-haplotypic for each sample within replicate.
